# Low serum complement C3 levels are associated with all-cause mortality in Chinese centenarians: a prospective cohort study

**DOI:** 10.3389/fimmu.2026.1911948

**Published:** 2026-09-09

**Authors:** WeiGuang Zhang, Yuting Duan, Zhe Li, Miaomiao Liu, Qiushi Wang, Yang Wang, Zehao Zhang, Zeyu Qu, Qiao Zhu, Yali Zhao, Miao Liu, Yao He, Qing Luo, Jie Zhang, Yizhi Chen

**Affiliations:** 1 Department of Nephrology, Hainan Hospital of Chinese PLA General Hospital, Hainan Province Flexible Talent Attraction Collaborative Innovation Center, Sanya, China; 2 Senior Department of Nephrology, Chinese PLA General Hospital, State Key Laboratory of Kidney Diseases, National Clinical Research Center for Kidney Diseases, Beijing Key Laboratory of Medical Devices and Integrated Traditional Chinese and Western Drug Development for Severe Kidney Diseases, Beijing Key Laboratory of Digital Intelligent TCM for the Prevention and Treatment of Pan-vascular Diseases, Key Disciplines of National Administration of Traditional Chinese Medicine (ZYYZDXK-2023310), Innovation Team and Talents Cultivation Program of National Administration of Traditional Chinese Medicine (ZYYCXTD-D-202402), Beijing, China; 3 Department of Geriatric Medicine, The Affiliated Hospital of Qingdao University, Qingdao, China; 4 Luoyang Key Laboratory of Clinical Multiomics and Translational Medicine, Henan Key Laboratory of Rare Diseases, Endocrinology and Metabolism Center, The First Affiliated Hospital, and College of Clinical Medicine of Henan University of Science and Technology, Luoyang, China; 5 Department of Laboratory Medicine, Hainan Hospital of Chinese PLA General Hospital, Sanya, China; 6 Central Laboratory, Hainan Hospital of Chinese PLA General Hospital, Sanya, China; 7 Department of Anti-Nuclear, Biological, Chemical Medicine, the Graduate School of Chinese PLA General Hospital, Beijing, China; 8 Institute of Geriatrics, the Second Medical Center of Chinese PLA General Hospital, Beijing Key Laboratory of Geriatric Comorbidity, National Clinical Research Center for Geriatrics Diseases, Beijing, China; 9 The Second School of Clinical Medicine, Southern Medical University, Guangzhou, China; 10 Hainan Medical University, Haikou, China; 11 Sanya Nephrology Medical Quality Control Center, Sanya, China

**Keywords:** all-cause mortality, centenarian, complement C3, complement C4, immunity

## Abstract

**Background:**

The complement system plays an important role in innate immunity and age-related inflammation, but its prognostic value in centenarians remains unclear. This study aimed to examine the associations between serum complement C3 and C4 levels and all-cause mortality among centenarians.

**Methods:**

We studied centenarians in Hainan Island, China. We used multivariable-adjusted restricted cubic splines, Cox proportional hazards models, and Kaplan–Meier survival analysis to assess the relationships between serum C3 and C4 levels and all-cause mortality.

**Results:**

A total of 906 centenarians were included in the final analysis. During a median follow-up of 30.1 months, 838 (92.5%) participants died. There was a significant inverse linear relationship between serum C3 levels and all-cause mortality (*P* = 0.031). Compared with the highest C3 quartile (Q4), individuals in the lowest quartile (Q1) had an increased risk of all-cause mortality (hazard ratio = 1.337; 95% confidence interval: 1.092–1.635; *P* = 0.005), whereas C4 levels showed no significant relationship with mortality (*P* > 0.05). When we used Q1 (0.86 g/L) as a cut-off, centenarians with C3 levels below this threshold also showed a higher mortality risk than those above this threshold (hazard ratio = 1.250; 95% confidence interval: 1.063–1.470; *P* = 0.007). A Kaplan–Meier analysis further indicated a shorter survival time in the lowest C3 quartile (Q1 vs. Q4: 26 vs. 34 months, *P* = 0.002). In contrast, C4 levels were not significantly associated with survival.

**Conclusion:**

Serum complement C3 levels, but not C4 levels, might be associated with the risk of all-cause mortality in centenarians. However, because all centenarians were recruited from Hainan Island, external validation in other regions and ethnic populations is warranted.

## 1 Introduction

With demographics shifting toward an older global population, research on the underlying mechanisms of aging and potential countermeasures is of paramount importance (1). This process is fundamentally defined by a deterioration in tissue and organ function, which manifests as an increased risk of numerous age-related diseases, thereby causing considerable societal burdens. Centenarians serve as a natural model of successful aging (2) and are unique by largely evading or delaying major diseases and associated disabilities to achieve exceptional longevity with preserved health (3). Therefore, studying centenarians provides invaluable insights for promoting healthy aging.

Within the aging organism, the immune system is a critical factor, exerting broad systemic effects. As an integral part of the innate immune system, the complement system is responsible not only for clearing pathogens but also for performing immunomodulatory functions. The complement system plays important roles in inflammatory processes, metabolism, apoptosis, and mitochondrial function (4). There is mounting evidence that the complement system plays a major role in age-related diseases, including Alzheimer’s disease (5), atherosclerosis (6), age-related macular degeneration (7), and osteoarthritis (8). Cao et al. showed that C3 and C4 could be used to develop a biological age model in Chinese Han adults with a median age of 34 years (9), suggesting a role in aging physiology.

Immunosenescence and inflammaging are central features of advanced aging, and the complement system represents a key interface between innate immune defense and inflammatory regulation. However, evidence regarding complement profiles and survival in centenarians remains limited. Previous studies have suggested that complement C3 and C4 levels differ in exceptionally long-lived individuals. Fu et al. reported that the longevity of centenarians was negatively correlated with C3 and C4 levels (10), but their study was cross-sectional and did not include longitudinal follow-up or mortality endpoints. Therefore, whether baseline complement levels can predict subsequent survival among centenarians remains unclear.

To address this knowledge gap, this study used data from the China Hainan Centenarian Cohort Study (CHCCS) with prospective follow-up to investigate the associations between serum C3 and C4 levels and all-cause mortality. We hypothesized that low serum C3 levels are associated with an increased risk of mortality, possibly reflecting impaired innate immune reserve and chronic inflammatory imbalance, whereas C4 does not have independent prognostic value because of its more restricted role in the classical and lectin complement pathways.

## 2 Methods

### 2.1 Study population

This study used data from the CHCCS (10), a prospective cohort study of centenarians conducted in Hainan Province, China. The cohort was established between July 2014 and December 2016, and the median follow-up period was 30.1 months. Death records were verified by a rigorous, triple-confirmation process as follows: (a) linkage to the National Cause of Death System (China CDC); (b) certification by civil affairs authorities; and (c) next-of-kin telephone interviews. Furthermore, monthly vital status verification was stipulated by the Hainan Civil Affairs Bureau via its pension program for octogenarians and older, allowing for real-time survival tracking. The study protocol was approved by the Ethics Committee of the Chinese People’s Liberation Army General Hospital (Approval No. 301HNLL-2016-01) and was performed in accordance with the Declaration of Helsinki. Before enrollment, trained investigators explained the study objectives, procedures, potential risks, and confidentiality protections to all participants and/or their legal representatives. Written informed consent was obtained from each participant or from a legally authorized representative when necessary.

### 2.2 Covariates

Standardized data capture encompassed the following: demographics, including age, sex, ethnicity, marital status, education, height, and weight; lifestyle, including tobacco and alcohol use patterns; and the medical history, including physician-diagnosed hypertension, diabetes mellitus, and coronary heart disease. Case definitions required a self-reported diagnosis or relevant medication use. Hypertension was confirmed by systolic/diastolic blood pressure ≥ 140/90 mmHg, and diabetes mellitus was defined in accordance with World Health Organization 1999 criteria (11). Anthropometrics (weight and height) followed standardized protocols with body mass index (BMI) calculated as weight/height^2^ (kg/m^2^).

Venous blood sampling was performed by trained nurses. Serum levels of C3 and C4 were quantified using an immune scattering turbidimetry approach (Siemens Healthineers, Germany) with a BNII automated protein analyzer (BNII; Siemens AG, Munich, Germany). All assays were performed at the Laboratory Department of Hainan Hospital of the PLA General Hospital.

### 2.3 Statistical methods

Before statistical analysis, the normality and homogeneity of variance of all data were assessed. Continuous variables with a normal distribution are presented as the mean ± standard deviation, and those with a skewed distribution are reported as the median and interquartile range. Group comparisons for normally distributed data were performed using Student’s t-test or one-way analysis of variance. Non-parametric data were analyzed using the Mann–Whitney U test or the Kruskal–Wallis H test. Categorical variables, which are expressed as numbers and percentages, were compared using the χ^2^ test.

Complement C3 and C4 levels were categorized into quartiles according to their respective interquartile range cut-off values. Complement C3 levels were divided into four groups: lowest (Q1: ≥ 0.480 to < 0.860 g/L), second lowest (Q2: ≥ 0.860 to < 0.975 g/L), second highest (Q3: ≥ 0.975 to < 1.110 g/L), and highest (Q4: ≥ 1.110 to ≤ 1.900 g/L). Additionally, C3 was dichotomized into two groups of Q1 (< 0.860 g/L) and Q2–Q4 (≥ 0.860 g/L) on the basis of the 25th percentile value. Similarly, C4 levels were categorized into four quartiles: lowest (Q1: ≥ 0.082 to < 0.187 g/L), second lowest (Q2: ≥ 0.187 to < 0.229 g/L), second highest (Q3: ≥ 0.229 to < 0.279 g/L), and highest (Q4: ≥ 0.279 to ≤ 0.788 g/L). C4 levels were also grouped into two categories according to the 50th percentile value: Q1–Q2 (< 0.229 g/L) and Q3–Q4 (≥ 0.229 g/L).

The associations between serum C3 and C4 levels and all-cause mortality were assessed using restricted cubic spline (RCS) models and Cox proportional hazards models. The proportional hazards assumption was evaluated using Scaled Schoenfeld residuals before a Cox regression analysis. RCS models were fitted using the rms package (with 4 knots), while Cox and Kaplan–Meier analyses were performed with the survival package. Kaplan–Meier curves depict time-to-event distributions, and survival differences were evaluated by log-rank testing. Subgroup analyses were performed using multivariable Cox models to examine whether the associations between serum C3 and C4 levels and all-cause mortality differed across predefined subgroups. Interaction effects were assessed by including multiplicative interaction terms in the Cox models. Hazard ratios (HRs) were estimated per 0.1 g/L increase in serum C3 or C4 levels.

A mediation analysis was performed to quantify whether BMI, IgG, IgM, IgA, IgE, total light chain kappa, and total light chain lambda mediated the association between serum C3 levels and all-cause mortality. The natural direct effect, natural indirect effect, total effect, and proportion mediated were estimated using a counterfactual framework with the CMA verse package, and 95% confidence intervals (CIs) were calculated from 1,000 bootstrap resamples. The models were adjusted for the same covariates as the primary multivariable Cox model, and these analyses were considered exploratory.

The primary models were adjusted for 17 prespecified covariates comprising age, sex, ethnicity, marital status, BMI, education, smoking status, alcohol consumption, diabetes mellitus, hypertension, coronary heart disease, immunoglobulin (Ig) G, IgM, IgA, IgE, total light chain kappa, and total light chain lambda. To minimize potential multicollinearity or overadjustment because of the biological correlation between C3 and C4 levels, C4 was not included as a covariate in the primary multivariable models evaluating C3, and C3 was not included as a covariate in the primary multivariable models evaluating C4. In sensitivity analyses, we additionally fitted mutually adjusted models, in which C4 was added to the C3 models and C3 was added to the C4 models. A two-sided *P* < 0.05 was considered statistically significant. All analyses were performed using R software (version 4.3.3).

## 3 Results

### 3.1 Baseline characteristics and follow-up

As detailed in our recent study (12), we excluded individuals with monoclonal gammopathy, other hematological disorders, or incomplete data. The final analysis comprised 906 participants. During a median follow-up of 30.1 months, 838 (92.5%) participants died. **Table 1** shows the baseline characteristics stratified by C3 quartiles. Comparisons across quartiles showed significant differences in the proportion of sex, BMI, and IgG, IgA, and C4 levels (all *P* < 0.05). Notably, centenarians with lowest C3 levels had a relatively higher proportion of male participants than their counterparts with highest C3 levels (23.1% in Q1 vs 13.0% in Q4, *P* = 0.015). Only two centenarians had C3 levels above the clinical upper limit, whereas approximately one-third had levels below the clinical lower limit; the remainder fell within the normal range. In contrast, > 90% of centenarians had C4 levels within the normal clinical range.

**Table 1:** Characteristics of the study population based on C3 quartiles at baseline.

Characteristics	ALL	Q1 [0.48,0.86)	Q2 [0.86,0.975)	Q3 [0.975,1.11)	Q4 [1.11,1.9]	*P*
N	906	221	232	214	239	
Age, years	102.00 (101.00, 104.00)	102.00 (101.00, 104.00)	102.00 (101.00, 105.00)	102.00 (101.00, 104.00)	102.00 (101.00, 104.00)	0.187
Sex						
Male, %	170(18.8)	51(23.1)	52(22.4)	36(16.8)	31(13.0)	0.015
Female, %	736 (81.2)	170 (76.9)	180 (77.6)	178 (83.2)	208 (87.0)	
Follow-up time, months	30.10 (14.80, 53.22)	26.10 (11.90, 44.20)	29.95 (15.80, 53.50)	31.25 (14.62, 51.38)	34.50 (16.05, 58.20)	0.016
Death, %	838 (92.5)	211 (95.5)	217 (93.5)	199 (93.0)	211 (88.3)	0.025
Body mass index, kg/m^2^	18.15 (16.22, 20.08)	17.78 (15.79, 19.33)	17.86 (16.04, 19.68)	18.22 (16.45, 20.30)	18.77 (16.69, 20.65)	<0.001
Ethnicity						
Han, %	804 (88.7)	199 (90.0)	204 (87.9)	189 (88.3)	212 (88.7)	0.904
Other, %	102 (11.3)	22 (10.0)	28 (12.1)	25 (11.7)	27 (11.3)	
Marital status						
Married, %	96 (10.6)	26 (11.8)	28 (12.1)	21 (9.8)	21 (8.8)	0.612
Separation/Divorce/ Widowhood, %	810 (89.4)	195 (88.2)	204 (87.9)	193 (90.2)	218 (91.2)	
Education						
Illiterate, %	824 (90.9)	193 (87.3)	216 (93.1)	194 (90.7)	221 (92.5)	0.066
Elementary school, %	63 (7.0)	25 (11.3)	10 (4.3)	13 (6.1)	15 (6.3)	
Junior high school and above, %	19 (2.1)	3 (1.4)	6 (2.6)	7 (3.3)	3 (1.3)	
Smoker						
Never, %	806 (89.0)	195 (88.2)	209 (90.1)	186 (86.9)	216 (90.4)	0.507
Past, %	68 (7.5)	19 (8.6)	12 (5.2)	21 (9.8)	16 (6.7)	
Now, %	32 (3.5)	7 (3.2)	11 (4.7)	7 (3.3)	7 (2.9)	
Alcohol user						
Never, %	746 (82.3)	182 (82.4)	193 (83.2)	174 (81.3)	197 (82.4)	0.663
Past, %	71 (7.8)	21 (9.5)	19 (8.2)	17 (7.9)	14 (5.9)	
Now, %	89 (9.8)	18 (8.1)	20 (8.6)	23 (10.7)	28 (11.7)	
Hypertension, %	670 (74.0)	153 (69.2)	174 (75.0)	162 (75.7)	181 (75.7)	0.331
Diabetes mellitus, %	82 (9.1)	12 (5.4)	26 (11.2)	17 (7.9)	27 (11.3)	0.085
Coronary heart disease, %	40 (4.4)	11 (5.0)	13 (5.6)	10 (4.7)	6 (2.5)	0.387
IgG, g/L	15.70 (13.60, 18.10)	14.70 (12.80, 17.40)	16.00 (14.00, 17.92)	16.05 (14.00, 18.17)	16.20 (13.40, 18.65)	0.001
IgM, g/L	1.02 (0.71, 1.40)	0.98 (0.72, 1.39)	1.04 (0.71, 1.34)	0.99 (0.70, 1.37)	1.06 (0.69, 1.49)	0.568
IgA, g/L	3.33 (2.53, 4.25)	3.29 (2.43, 4.13)	3.24 (2.53, 4.25)	3.27 (2.45, 4.12)	3.55 (2.70, 4.50)	0.033
IgE, IU/mL	273.00 (81.50, 814.75)	262.00 (72.00, 690.00)	319.00 (100.75, 1052.50)	300.50 (88.50, 923.00)	227.00 (69.50, 710.50)	0.147
Kappa, g/L	413.00 (354.00, 478.00)	401.00 (351.00, 463.00)	414.00 (365.75, 470.25)	413.50 (345.50, 477.00)	427.00 (356.50, 496.50)	0.276
Lambda, g/L	209.00 (178.25, 249.00)	203.00 (171.00, 241.00)	209.00 (181.00, 247.50)	211.00 (182.25, 245.00)	211.00 (180.00, 254.50)	0.174
C3 Continuous, g/L	0.98 (0.86, 1.11)	0.78 (0.71, 0.82)	0.92 (0.89, 0.95)	1.03 (1.01, 1.06)	1.23 (1.16, 1.35)	<0.001
C3 Categories						
Low group <0.9 g/L, %	296 (32.7)	221 (100.0)	75 (32.3)	0 (0.0)	0 (0.0)	<0.001
Normal group [0.9-1.8] g/L, %	608 (67.1)	0 (0.0)	157 (67.7)	214 (100.0)	237 (99.2)	
High group >1.8 g/L, %	2 (0.2)	0 (0.0)	0 (0.0)	0 (0.0)	2 (0.8)	
C4 Continuous, g/L	0.23 (0.19, 0.28)	0.19 (0.16, 0.24)	0.22 (0.18, 0.26)	0.24 (0.20, 0.29)	0.27 (0.23, 0.33)	<0.001
C4 Categories						
Low group <0.1 g/L, %	4 (0.4)	2 (0.9)	0 (0.0)	1 (0.5)	1 (0.4)	<0.001
Normal group [0.1-0.4] g/L, %	855 (94.4)	214 (96.8)	228 (98.3)	205 (95.8)	208 (87.0)	
High group >0.4 g/L, %	47 (5.2)	5 (2.3)	4 (1.7)	8 (3.7)	30 (12.6)	

### 3.2 RCS analyses of the associations between serum C3 and C4 levels and all-cause mortality

**Figure 1:**
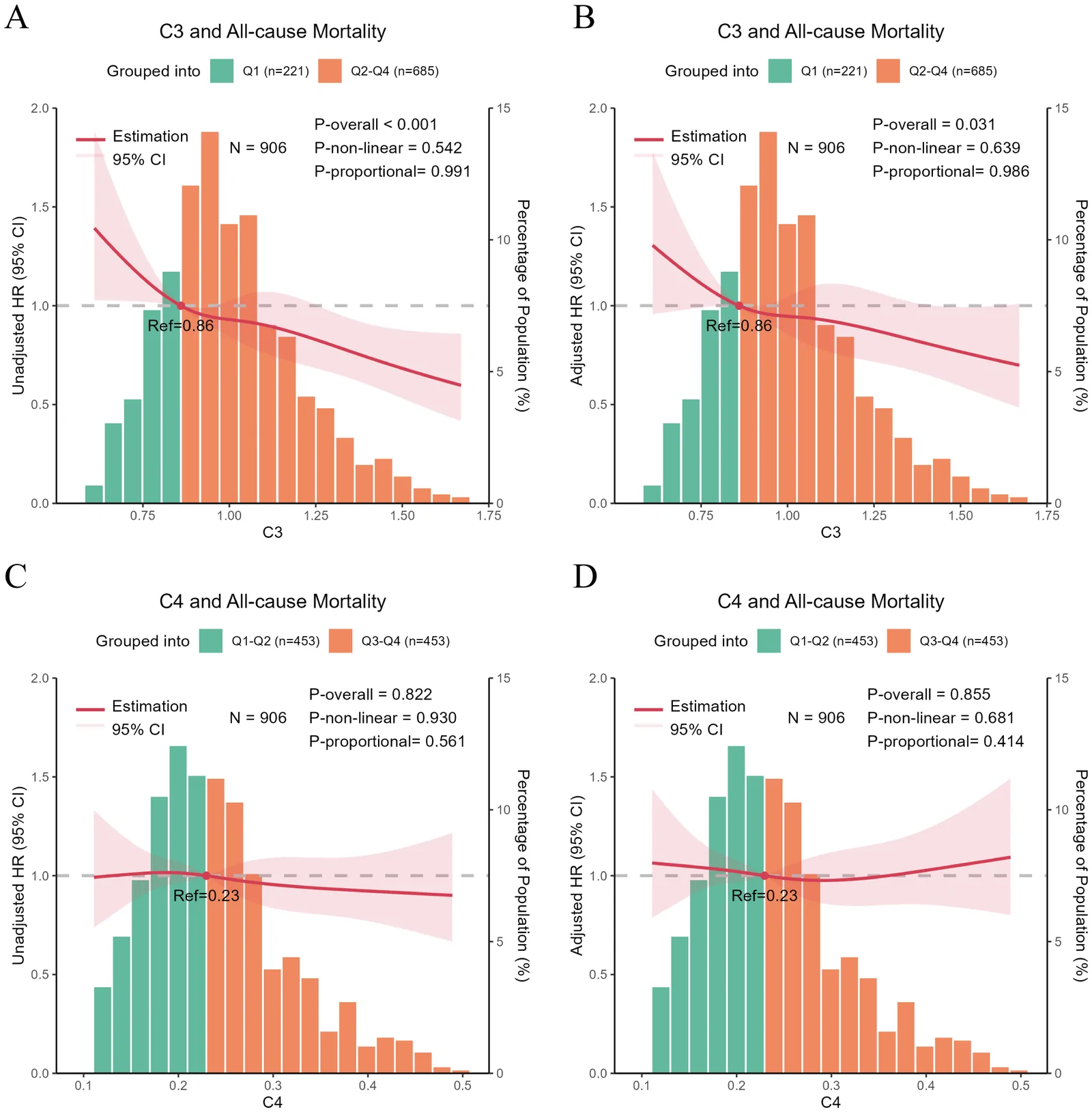
Restricted cubic spline analyses of serum C3 and C4 levels with all-cause mortality. Panels **(A, B)** show the associations between serum C3 and all-cause mortality in unadjusted model and the primary multivariable-adjusted model without C4 adjustment. Panels **(C, D)** show the associations between serum C4 and all-cause mortality in unadjusted model and the primary multivariable-adjusted model without C3 adjustment. The primary multivariable models were adjusted for age, sex, ethnicity, marital status, body mass index, education, smoking status, alcohol consumption, diabetes mellitus, hypertension, coronary heart disease, IgG, IgM, IgA, IgE, total light chain kappa, and total light chain lambda. The solid line represents the estimated hazard ratio, and the shaded area represents the 95% confidence interval. Reference values were 0.86 g/L (P25) for C3 and 0.229 g/L (P50) for C4.

To address potential multicollinearity and overadjustment between C3 and C4, we used RCS models without mutual adjustment between the two complement components as the primary analysis. Regarding C3, the unadjusted model showed a significant inverse association between C3 levels and all-cause mortality (*P*-overall < 0.001; **Figure 1A**). This association remained significant in the primary multivariable-adjusted model without C4 adjustment (*P*-overall = 0.031, *P*-nonlinear = 0.639; **Figure 1B**). In the sensitivity model with additional adjustment for C4, the association between C3 and mortality remained similar (*P*-overall = 0.018, *P*-nonlinear = 0.657; **Supplementary Figure 1A**). Regarding C4, no significant association between C4 levels and all-cause mortality was observed in the unadjusted model (*P*-overall = 0.822; **Figure 1C**), the primary multivariable-adjusted model without adjustment for C3 (*P*-overall = 0.855; **Figure 1D**), or the sensitivity model with additional adjustment for C3 (*P*-overall = 0.628; **Supplementary Figure 1B**).

### 3.3 Univariate and multivariable Cox analyses of the associations between serum C3 and C4 levels and all-cause mortality

Scaled Schoenfeld residuals were plotted against the follow-up time to assess the proportional hazards assumption in the primary multivariable Cox model evaluating C3 and all-cause mortality (**Supplementary Figure 2**). The global test showed no significant violation of the proportional hazards assumption (*P* = 0.265), and C3 showed no significant time-dependent effect (*P* = 0.99), supporting the overall validity of the proportional hazards assumption. Although the Kaplan–Meier curves crossed during the early follow-up period, however, the proportional hazards assumption was not violated according to Scaled Schoenfeld residuals. Univariate and multivariable Cox analyses and Kaplan–Meier survival analyses were then performed.

Each 0.1 g/L increase in C3 levels was associated with a reduced mortality risk in the univariable model (HR = 0.936, 95% CI: 0.906–0.967, *P* < 0.001) and the primary multivariable-adjusted model without adjustment for C4 (HR = 0.952, 95% CI: 0.920–0.986, *P* = 0.005) (**Table 2**). Using the highest C3 quartile as the reference, participants in the lowest quartile had the highest mortality risk in the primary multivariable-adjusted model (HR = 1.337, 95% CI: 1.092–1.635, *P* = 0.005). When C3 levels were dichotomized according to the first quartile cut-off point, participants with C3 levels < 0.86 g/L also had a higher mortality risk than those with C3 levels ≥ 0.86 g/L (HR = 1.250, 95% CI: 1.063–1.470, *P* = 0.007). In contrast, serum C4 levels were not significantly associated with all-cause mortality in the primary multivariable-adjusted Cox models without adjustment for C3. In the sensitivity Cox analyses with mutual adjustment between C3 and C4, the results were generally consistent with the primary analyses. After additional adjustment for C4, the association between C3 levels and mortality remained significant, whereas C4 levels remained non-significantly associated with mortality after additional adjustment for C3 (**Supplementary Table 1**).

**Table 2:** Univariate and multivariate Cox proportional hazards analyses of the associations of C3 and C4 with all-cause mortality in centenarians.

	Number	Univariate analyses		Multivariate-adjusted analyses	
Characteristics		HR	*P*	HR	*P*
C3					
Continuous, per 0.1 g/L	906	0.936 (0.906,0.967)	<0.001	0.952 (0.920,0.986)	0.005
C3 grouped by Interquartile Value (g/L)					
Q1 [0.48,0.86)	221	1.459 (1.204,1.767)	<0.001	1.337 (1.092,1.635)	0.005
Q2 [0.86,0.975)	232	1.166 (0.964,1.409)	0.113	1.072 (0.879,1.307)	0.494
Q3 [0.975,1.11)	214	1.201 (0.989,1.458)	0.065	1.142 (0.937,1.392)	0.188
Q4 [1.11,1.9]	239	1 (Reference)		1 (Reference)	
*P* for trend			<0.001		0.015
Q1 [0.48,0.86)	221	1.308 (1.119,1.530)	<0.001	1.250 (1.063,1.470)	0.007
Q2-Q4 [0.86,1.9]	685	1 (Reference)		1 (Reference)	
C4					
Continuous, per 0.1 g/L	906	0.964 (0.889,1.045)	0.369	0.995 (0.914,1.084)	0.913
C4 grouped by Interquartile Value (g/L)					
Q1 [0.082,0.187)	225	1.075 (0.888,1.301)	0.460	1.016 (0.834,1.238)	0.875
Q2 [0.187,0.229)	228	1.068 (0.883,1.292)	0.497	1.013 (0.834,1.230)	0.898
Q3 [0.229,0.279)	223	1.008 (0.830,1.224)	0.936	0.945 (0.776,1.150)	0.572
Q4 [0.279,0.788]	230	1 (Reference)		1 (Reference)	
*P* for trend			0.375		0.711
Q1-Q2 [0.082,0.229)	453	1.067 (0.932,1.222)	0.347	1.044 (0.908,1.200)	0.545
Q3-Q4 [0.229,0.788]	453	1 (Reference)		1 (Reference)	

Multivariate-adjusted Analyses: Adjusted for age, sex, ethnicity, marital status, body mass index (BMI), education, smoking status, alcohol consumption, diabetes mellitus, hypertension, and coronary artery disease, IgG, IgM, IgA, IgE, total light chain kappa, total light chain lambda. When taking C3 or C4 as the predictor, C4 or C3 was not included in the multivariate -adjusted analyses.

### 3.4 Kaplan–Meier survival analyses stratified by serum C3 and C4 levels

**Figure 2:**
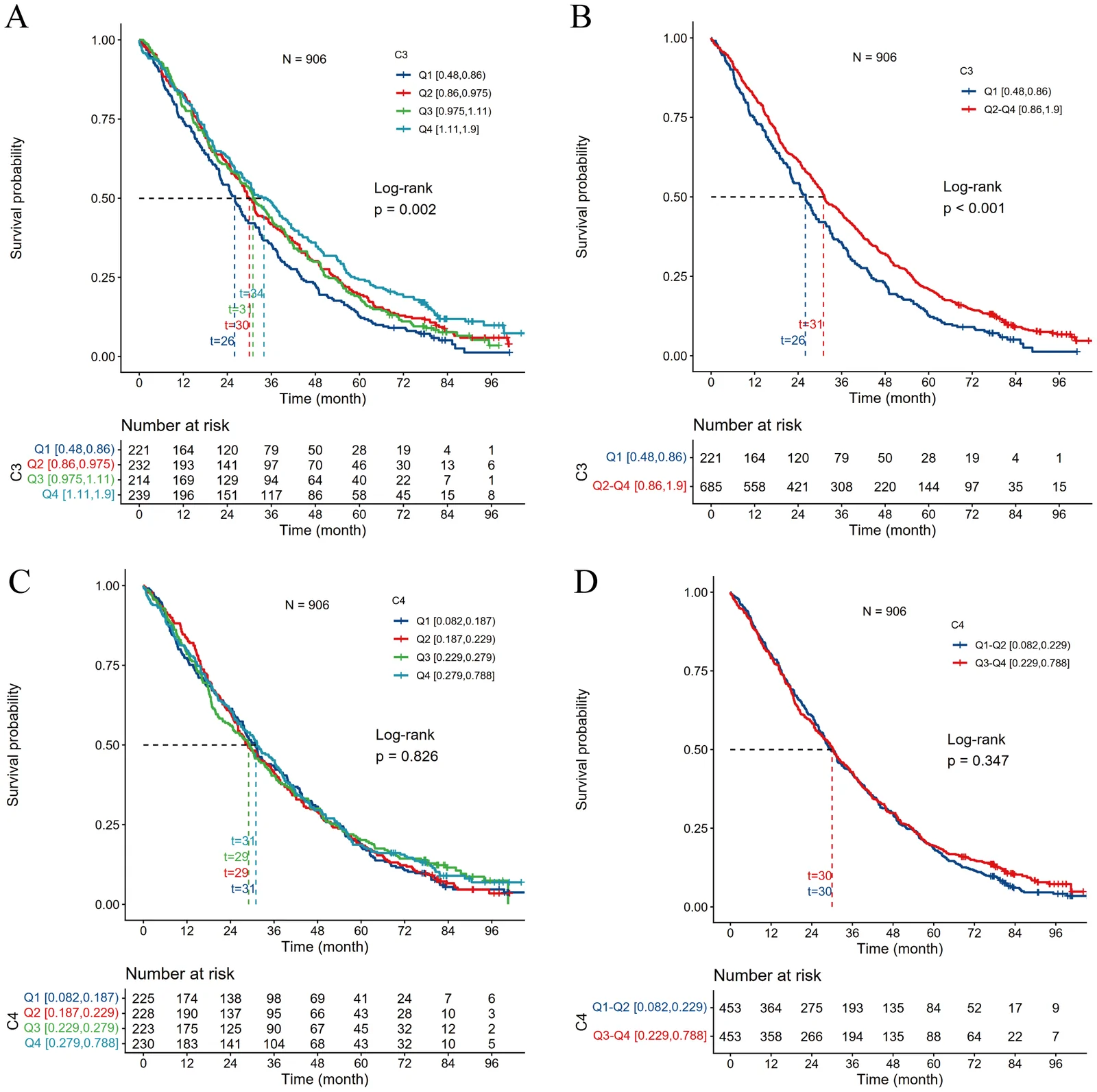
Kaplan-Meier survival curves and log-rank tests for the associations of all-cause mortality with serum C3 and C4 levels. Kaplan-Meier survival curves revealed significant associations of lower C3 levels with reduced survival time. The median survival time was significantly shorter in centenarians with the lowest quartile of C3 than in those with the highest quartile of C3 (26 months vs. 34 months, *P* = 0.002) **(A)**. The median survival time was significantly shorter in centenarians with complement C3 levels below the first quartile cut-off point (P25 < 0.86g/L) compared to those with C3 levels above this threshold (≥ 0.86g/L) (26 months vs. 31 months, *P* < 0.001) **(B)**. In contrast, the Kaplan-Meier curves confirmed that C4 levels were not significantly associated with increased all-cause mortality **(C, D)**.

The median survival time was significantly shorter in centenarians in the lowest quartile of C3 than in those in the highest quartile (26 months vs. 34 months, *P* = 0.002) (**Figure 2A**). When the participants were stratified by the first quartile cut-off (25th percentile, 0.86 g/L), those with C3 levels < 0.86 g/L also had a significantly shorter median survival than those with C3 levels ≥ 0.86 g/L (26 months vs. 31 months, *P* < 0.001) (**Figure 2B**). Although the Cox model does not directly estimate the reduction in survival time per 0.1 g/L decrease in C3 levels, the Kaplan–Meier analysis showed clinically meaningful differences in the median survival time. In short, the median survival time was 8 months shorter in the lowest C3 quartile than in the highest C3 quartile and 5 months shorter in centenarians with C3 levels < 0.86 g/L than in those with C3 levels ≥ 0.86 g/L. In contrast, Kaplan–Meier curves showed that C4 levels were not significantly associated with all-cause mortality (**Figures 2C, D**).

### 3.5 Subgroup analyses of factors affecting the associations between C3 and C4 levels and mortality

**Figure 3:**
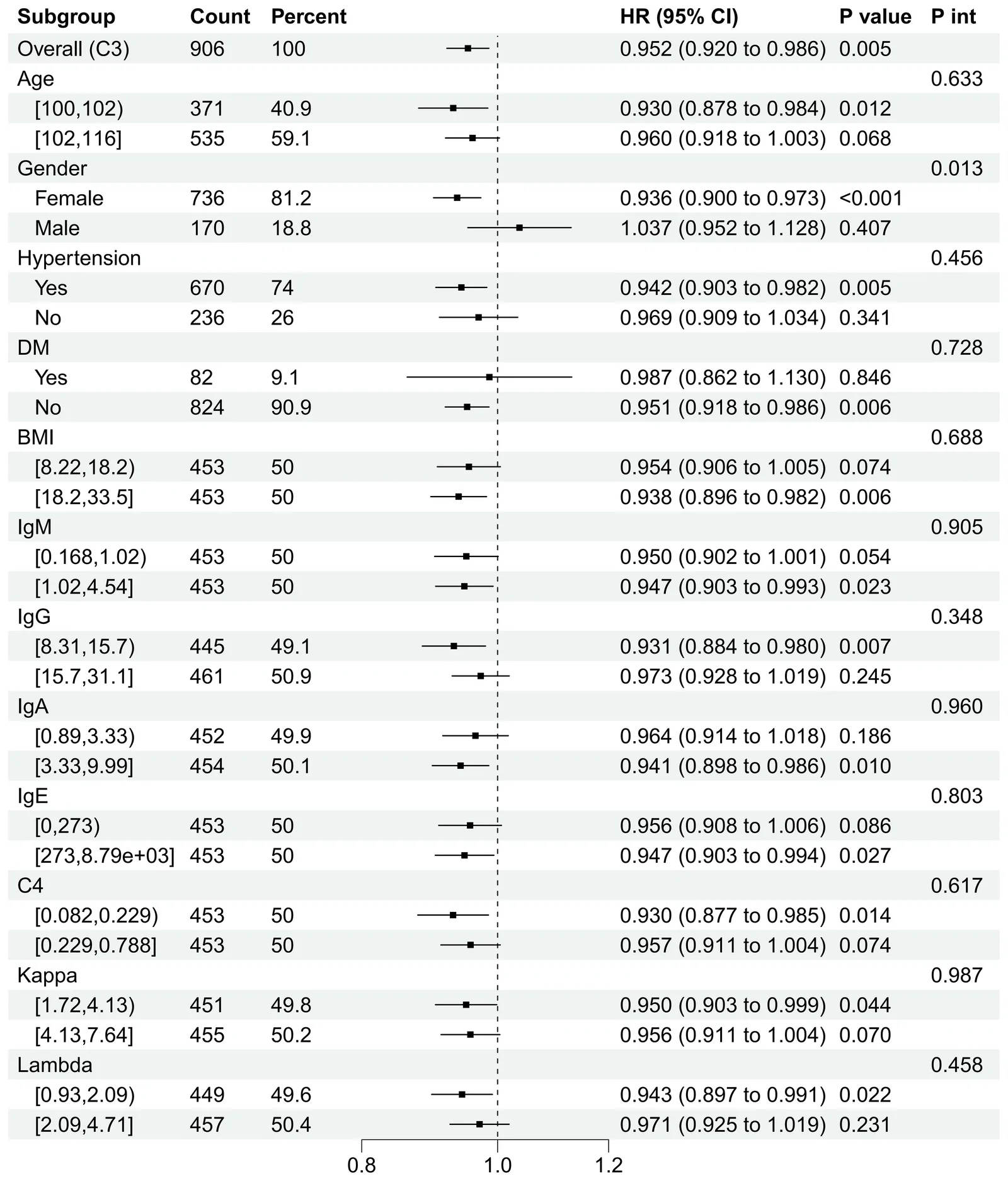
Forest plots of adjusted hazard ratios for serum C3 levels across predefined subgroups. Each 0.1 g/L increment was associated with a significantly reduced risk of all-cause mortality in the overall population (adjusted HR = 0.952, 95% CI: 0.920–0.986; *P* = 0.005). A significant interaction was detected only for sex (*P* for interaction = 0.013), indicating a stronger inverse association in women. No other subgroup interactions reached statistical significance. The primary multivariable models were adjusted for age, sex, ethnicity, marital status, body mass index, education, smoking status, alcohol consumption, diabetes mellitus, hypertension, coronary heart disease, IgG, IgM, IgA, IgE, total light chain kappa, and total light chain lambda.

A significant interaction was observed for sex (*P* for interaction = 0.013) (**Figure 3**). An inverse association between C3 levels and mortality was observed in women (HR = 0.936, 95% CI: 0.900–0.973, *P* < 0.001), whereas no significant association was observed in men (HR = 1.037, 95% CI: 0.952–1.128, *P* = 0.407). No significant interactions were observed in other subgroups, including age, hypertension, diabetes mellitus, BMI, IgM, IgG, IgA, IgE, C4, total light chain kappa, and total light chain lambda. The association between C4 levels and all-cause mortality was generally consistent in predefined subgroups, and no significant interactions were detected for age, sex, hypertension, diabetes mellitus, BMI, IgM, IgG, IgA, IgE, C3, total light chain kappa, or total light chain lambda (**Supplementary Figure 3**).

### 3.6 Exploratory mediation analysis

BMI showed a significant partial mediating effect of the association between C3 levels and all-cause mortality, with a proportion mediated of 22.8% (95% CI: 9.1%–55.4%, P < 0.001), which suggested partial mediation (**Supplementary Figure 4**). In contrast, IgG, IgM, IgA, IgE, total light chain kappa, and total light chain lambda showed no significant mediating effects.

## 4 Discussion

To the best of our knowledge, this study is the first to suggest an inverse and linear association between serum complement C3 levels and all-cause mortality in centenarians. This association remained robust after comprehensive adjustment for demographics, comorbidities, and other immunological parameters, and was notably absent for C4 levels. The distinct predictive value of C3 suggests that it has unique biological significance for survival in exceptionally long-lived individuals. Mutual adjustment may introduce multicollinearity or overadjustment because C3 and C4 are biologically related. Therefore, we used models without mutual adjustment in the primary analyses and used mutually adjusted models in sensitivity analyses. The association of low C3 levels and increased mortality was consistent in the primary and sensitivity analyses.

The complement system is a crucial component of innate immune defense, serving as an important driver against infection and inflammation (11, 12). In our previous study, we found that in centenarians, lower IgM levels and higher total light chain levels were associated with increased all-cause mortality (13, 14). These results suggest that centenarians show specialized immune functions and regulatory pathways that contribute to their longevity. Fu et al. found that the lifespan of centenarians was inversely related to their C3 and C4 levels compared with an 80-year-old population (10). However, their study was limited to cross-sectional data without follow-up endpoint events, which constrained its ability to establish causal relationships or trends over time. In contrast, our study overcame these limitations by incorporating endpoint events (mortality). We observed that lower C3 levels were associated with higher all-cause mortality, suggesting that maintaining C3 within the normal clinical range may be important for healthy aging.

Deficiencies in the complement system increase the risk of infection (15–17), and aberrant activation can cause or exacerbate a wide range of diseases (18). In a prospective study, Chen et al. enrolled 519 patients with Takayasu arteritis, among whom 406 had active disease. They found that elevated C3 levels were effective for evaluating the disease activity of Takayasu arteritis (19). However, Scurt et al. observed a higher risk of dialysis and death with lower C3 levels in 164 patients with antineutrophil antibody-associated vasculitis (20). Low C3/C4 levels were associated with increased mortality in 6800 patients who were antiphospholipid antibody positive (21). Jiang et al. observed a greater risk of clinical deterioration in 216 hospitalized patients with low C3 levels (22). Low C3 levels were associated with a greater incidence of cardiovascular disease in 5850 young and middle-aged healthy men with a follow-up of 18 years (23). Collectively, these studies are consistent with our finding that C3 levels < 0.86 g/L, which is marginally below the standard lower limit in our lab (0.9 g/L), were associated with a significantly increased mortality risk in centenarians. This pattern may reflect the dual role of C3 in modulating chronic inflammation: suboptimal concentrations potentially impair pathogen clearance capacity, whereas excessive levels may exacerbate tissue damage. From a clinical perspective, serum C3 levels are a routinely available and inexpensive laboratory marker. Our findings suggest that C3 levels < 0.86 g/L may help identify centenarians at a higher risk of mortality. Therefore, serum C3 measurement may be considered as part of comprehensive risk assessment in very old adults, especially in female centenarians. In individuals with markedly low C3 levels, closer clinical monitoring and evaluation of the nutritional status, inflammaging burden, infection susceptibility, and immune function may be warranted. Nevertheless, whether nutritional optimization or immune-modulating interventions can improve C3 levels or survival outcomes remains unknown and should be examined in future prospective interventional studies.

In this study, the baseline proportion of male sex was higher in centenarians with lowest C3 levels than in those with highest C3 levels (23.1% vs 13.0%). However, men constituted the minority of participants in the whole group (male 18.8% vs female 81.2%). In the survival analyses, significant association between lower serum C3 levels and high all-cause mortality was only observed in women but not in men. However, this finding should be interpreted cautiously. Women constituted the majority of our cohort, whereas the number of male centenarians was relatively small. Therefore, male-specific estimates were likely underpowered and statistically unreliable, regardless of whether the observed association in men was significant or not. Therefore, our findings regarding sex modification need to be confirmed in larger, sex-balanced cohorts. Women generally have a survival advantage in human populations (24). However, this advantage may be attenuated in the presence of markedly reduced C3 levels, which could be associated with impaired innate immune reserve, complement consumption, or reduce physiological resilience. In female centenarians, changes in sex hormone signaling and immunosenescence may affect immune homeostasis and inflammatory regulation (25). As the central component of the complement cascade, C3 integrates innate immune surveillance, pathogen clearance, and inflammaging signaling (16). Therefore, low circulating C3 levels may indicate compromised immune defense or systemic vulnerability in exceptionally old individuals, especially in women.

The complement system has three activation pathways: the classical pathway, the lectin pathway, and the alternative pathway (26). A simplified schematic diagram of the classical, lectin, and alternative complement activation pathways is shown in **Supplementary Figure 5** to facilitate interpretation of the distinct biological roles of C3 and C4. The three complement pathways share a critical dependency on C3 proteolysis as their central activation nexus, whereas C4 participates exclusively in the classical/lectin pathways (27). Upon activation, C3 is cleaved into effector fragments (e.g., C3a and C3b) that directly mediate pathogen opsonization, inflammaging signaling, and recruitment of phagocytes, contributing to host defense and modulation of systemic inflammation (28). In contrast, complement C4 primarily participates in the early stages of the classical and lectin pathways and is not involved in the alternative pathway. C3 lies downstream and is the central effector in all pathways. Therefore, circulating C3 levels may more sensitively reflect the overall degree of complement activation and systemic inflammaging burden in very old individuals. In aging populations, low-grade chronic inflammation (i.e., inflammaging) and altered innate immune responses are key features that affect survival (29, 30). In our centenarian cohort, lower C3 levels may have reflected impaired innate immune capacity, complement consumption, reduced physiological reserve, or a poor nutritional and metabolic status, which could have contributed to increased vulnerability to mortality. In contrast, C4, which is limited to selected complement activation pathways, may be less sensitive to these aging-associated survival-related processes than C3. This differential association may partially explain the distinct effects of C3 and C4 on all-cause mortality in centenarians.

C3 is biosynthesized not only in the liver but also within adipose tissue (by macrophages and endothelial cells), whereas C4 production occurs predominantly in hepatic tissues (31). Adipose tissue not only secretes cytokines that stimulate hepatic synthesis of C3 (32) but also potently activates C3 and other complement receptors (31). Centenarians represent a unique population characterized by a lower BMI and reduced abdominal fat (33). Therefore, the age-related decline in adipose tissue, particularly in women after menopause, could contribute to diminished C3 production. The reduced adipose tissue in centenarians could lead to diminished secretion of cytokines that stimulate C3 production, thereby maintaining C3 levels within a lower range. This possibility offers a potential explanation for the phenomenon of relatively low C3 levels but not low C4 levels. Our study showed that centenarians with low C3 levels had higher all-cause mortality than those with C3 levels within the normal range. This finding suggests that maintaining C3 levels within the normal clinical range might be a strategy for enhancing longevity in centenarians.

This study has several limitations. First, all of the participants were recruited from Hainan Island, which may have limited the generalizability of our findings because regional dietary habits, environmental exposures, lifestyle patterns, ethnic composition, and healthcare contexts may affect complement levels and survival. Future multicenter studies in geographically and ethnically diverse populations not only in China but throughout the world are required to validate the prognostic value of serum C3 levels. Second, only all-cause mortality was analyzed because reliable cause-specific death information was unavailable. Therefore, we could not determine whether cardiovascular, infectious, tumor-related, or other causes primarily drove the increased mortality associated with low C3 levels. Third, serum C3 and C4 levels were measured only once at baseline, precluding evaluation of longitudinal complement dynamics and differential effects of sustained reduced C3 levels versus a transient decline in C3 levels on all-cause mortality. Fourth, although the subgroup analysis suggested a significant C3 by sex interaction, the number of male centenarians was limited, which means that male-specific estimates were unreliable. Fifth, chronic diseases were adjusted for mainly as binary variables, and detailed information on the disease duration, medication use, treatment control, baseline blood pressure, blood glucose levels was not comprehensively available. Therefore, residual confounding cannot be fully excluded. Finally, causality cannot be established because this was an observational cohort study. Future studies with repeated complement measurements, adjudicated cause-of-death data, detailed clinical phenotyping, and larger sex-balanced multicenter cohorts are warranted.

## 5 Conclusion

This study showed that centenarians with lower complement C3 levels had a significantly higher all-cause risk of mortality, and this association was particularly pronounced in women. This finding underscores the critical role of C3 in survival of centenarians. Serum C3 levels may be useful as a biomarker for the risk of mortality in centenarians, especially in women, although further studies are required to confirm these findings and examine underlying mechanisms.

## Data Availability

The original contributions presented in the study are included in the article/**Supplementary Material**. Further inquiries can be directed to the corresponding authors.
