# Supplementary material for: Low serum complement C3 levels are associated with all-cause mortality in Chinese centenarians: a prospective cohort study
Source: Front Immunol. 2026 Sep 9;17:1911948. doi: 10.3389/fimmu.2026.1911948 (PMC13597452; doi:10.3389/fimmu.2026.1911948)
Supplement: Supplementary file 1 [file SupplementaryFile1.docx]

Supplementary Materials

## 1.1 Supplementary Figure 1

Supplementary Figure 1. Restricted cubic spline analyses with mutual adjustment between C3 and C4. Panel A shows the association between serum C3 and all-cause mortality after additional adjustment for C4. Panel B shows the association between serum C4 and all-cause mortality after additional adjustment for C3. In addition to the other complement component, the models were adjusted for age, sex, ethnicity, marital status, body mass index, education, smoking status, alcohol consumption, diabetes mellitus, hypertension, coronary heart disease, IgG, IgM, IgA, IgE, total light chain kappa, and total light chain lambda. The solid line represents the estimated hazard ratio, and the shaded area represents the 95% confidence interval.

Supplementary Figure 2. Scaled Schoenfeld residual plots for the primary multivariable Cox model of C3. Scaled Schoenfeld residuals were plotted against follow-up time to assess the proportional hazards assumption in the primary multivariable Cox model evaluating C3 and all-cause mortality. The global test showed no significant violation of the proportional hazards assumption (P = 0.265), and C3 showed no significant time-dependent effect (P = 0.99), supporting the overall validity of the proportional hazards assumption. The model was adjusted for age, sex, ethnicity, marital status, body mass index, education, smoking status, alcohol consumption, diabetes mellitus, hypertension, coronary heart disease, IgG, IgM, IgA, IgE, total light chain kappa, and total light chain lambda.

Supplementary Figure 3. Forest plot of adjusted hazard ratios for serum C4 and all-cause mortality across predefined subgroups. Hazard ratios were estimated per 0.1 g/L increase in serum C4. Subgroup analyses were performed according to age, sex, hypertension, diabetes mellitus, body mass index, IgM, IgG, IgA, IgE, C3, total light chain kappa, and total light chain lambda. No significant association between C4 and all-cause mortality was observed in the overall population or across predefined subgroups, and no significant interaction was detected. HR, hazard ratio; CI, confidence interval; BMI, body mass index; DM, diabetes mellitus. The model was adjusted for age, sex, ethnicity, marital status, BMI, education, smoking status, alcohol consumption, diabetes mellitus, hypertension, coronary heart disease, IgG, IgM, IgA, IgE, C3, total light chain kappa, and total light chain lambda.

Supplementary Figure 4. Exploratory mediation analyses of the association between serum C3 and all-cause mortality. Mediation analyses were performed using BMI, IgA, IgG, IgM, IgE, total light chain kappa, and total light chain lambda. BMI showed a significant partial mediating effect, whereas IgA, IgG, IgM, IgE, total light chain kappa, and total light chain lambda did not show significant mediation. PM indicates proportion mediated.

Supplementary Figure 5. Simplified schematic diagram of the three complement activation pathways. The classical, lectin, and alternative pathways are shown. All three pathways converge at C3 activation, highlighting C3 as the central component of complement amplification. In contrast, C4 mainly participates in the classical and lectin pathways and is not involved in the alternative pathway. This pathway difference may partly explain why C3, but not C4, showed independent prognostic value for all-cause mortality in centenarians. MBL, mannose-binding lectin; MAC, membrane attack complex.

Supplementary Table 1. Cox proportional hazards analyses with mutual adjustment between C3 and C4.

|  | Multivariate-adjusted Analyses | |
| --- | --- | --- |
|  | HR | *P* |
| C3 |  |  |
| Continuous, per 0.1 g/L | 0.944(0.909,0.980) | 0.003 |
| C3 grouped by Interquartile Value |  |  |
| Q1 [0.48,0.86) | 1.377(1.110,1.708) | 0.004 |
| Q2 [0.86,0.975) | 1.099(0.892,1.354) | 0.376 |
| Q3 [0.975,1.11) | 1.157(0.946,1.414) | 0.155 |
| Q4 [1.11,1.9] | 1 (Reference) |  |
| *P* for trend |  | 0.010 |
| Q1 [0.48,0.86) | 1.263(1.069,1.492) | 0.006 |
| Q2-Q4 [0.86,1.9] | 1 (Reference) |  |
| C4 |  |  |
| Continuous, per 0.1 g/L | 1.056(0.963,1.159) | 0.247 |
| C4 grouped by Interquartile Value |  |  |
| Q1 [0.082,0.187) | 0.904 (0.731,1.118) | 0.354 |
| Q2 [0.187,0.229) | 0.934(0.764,1.142) | 0.506 |
| Q3 [0.229,0.279) | 0.909(0.745,1.108) | 0.344 |
| Q4 [0.279,0.788] | 1 (Reference) |  |
| *P* for trend |  | 0.431 |
| Q1-Q2 [0.082,0.229) | 0.969(0.835,1.125) | 0.680 |
| Q3-Q4 [0.229,0.788] | 1 (Reference) |  |

For the C3 models, C4 was additionally included as a covariate. For the C4 models, C3 was additionally included as a covariate. All models were adjusted for age, sex, ethnicity, marital status, body mass index, education, smoking status, alcohol consumption, diabetes mellitus, hypertension, coronary heart disease, IgG, IgM, IgA, IgE, total light chain kappa, and total light chain lambda. Hazard ratios are presented with 95% confidence intervals.
